# Biceps sternalis: a Y-shaped muscle on the anterior chest wall

**DOI:** 10.1186/1749-8090-8-38

**Published:** 2013-03-07

**Authors:** Seyed Hadi Anjamrooz

**Affiliations:** 1 Department of Anatomical Sciences, School of Medicine, Kurdistan University of Medical Sciences, P.O. Box: 66177–13446, Sanandaj, Iran

**Keywords:** Sternalis muscle, Pectoralis major, Chest wall

## Abstract

The sternalis muscle is an accessory muscle located in the anterior thoracic region, which is relatively unfamiliar to clinicians and surgeons. To date, no data from the Iranian population have been published. Here, a rare case of a sternalis muscle is presented. In addition, this anomalous muscle was observed along with other visceral and vascular anomalies. This case is unique and provides significant information to radiologists, angiologists and surgeons seeking to apply safer interventions. It is also imperative for better interpretation of mammographic images and in reconstructive surgery.

## Background

The sternalis muscle is a very rare chest wall muscle variant with no apparent function and embryological origin. Familiarity with its appearance is essential for avoiding confusion with a wide range of lesions, such as breast carcinoma, extra-abdominal desmoid tumours, granular cell tumours, diabetic mastopathy, abscesses, hematomas, sclerosing adenositis, lymphadenitis, fat necrosis, and surgical scars. It also may lead to the misdiagnosis of breast cancer recurrence during post-treatment checkups. The presence of the sternalis muscle may alter the depth at which the internal mammary nodes are irradiated in cases of breast carcinoma, especially those lesions infiltrating the medial quadrants [1-3]. In addition, breast tissue extending deep to the sternalis muscle may be neglected during mastectomy [4]. This muscle has never been related to any clinical symptoms; however, its presence may evoke alterations in the electrocardiogram and may cause breast or chest asymmetry [5,6].

The sternalis muscle is occasionally observed as an unusual bulge in the medial aspect of craniocaudal mammograms. This muscle can also easily be identified using CT or MRI scans. CT shows a flat or oblique strap-like structure that overlies the pectoralis major muscle and is isodense with other anterior thoracic muscles. The incidental finding of a sternalis muscle in mammography, CT, or MRI studies must be documented in a patient’s clinical records, as it can be an ideal candidate for utilisation in plastic reconstruction of the head, neck, and anterior chest wall [3,7]. For example, use of a conjoined sternalis-pectoralis muscle flap in immediate tissue expander reconstruction after breastectomy has been proposed by reconstructive surgeons [8].

The existence of the sternalis has not been reported in Iranians. This is the first report in which the gross morphometry of a rare subtype of sternalis muscle, *biceps sternalis*, is described. The rarity was observed in the cadaver of an Iranian adult male. The muscle’s probable embryological origin is also discussed.

## Case presentation

**Figure 1:**
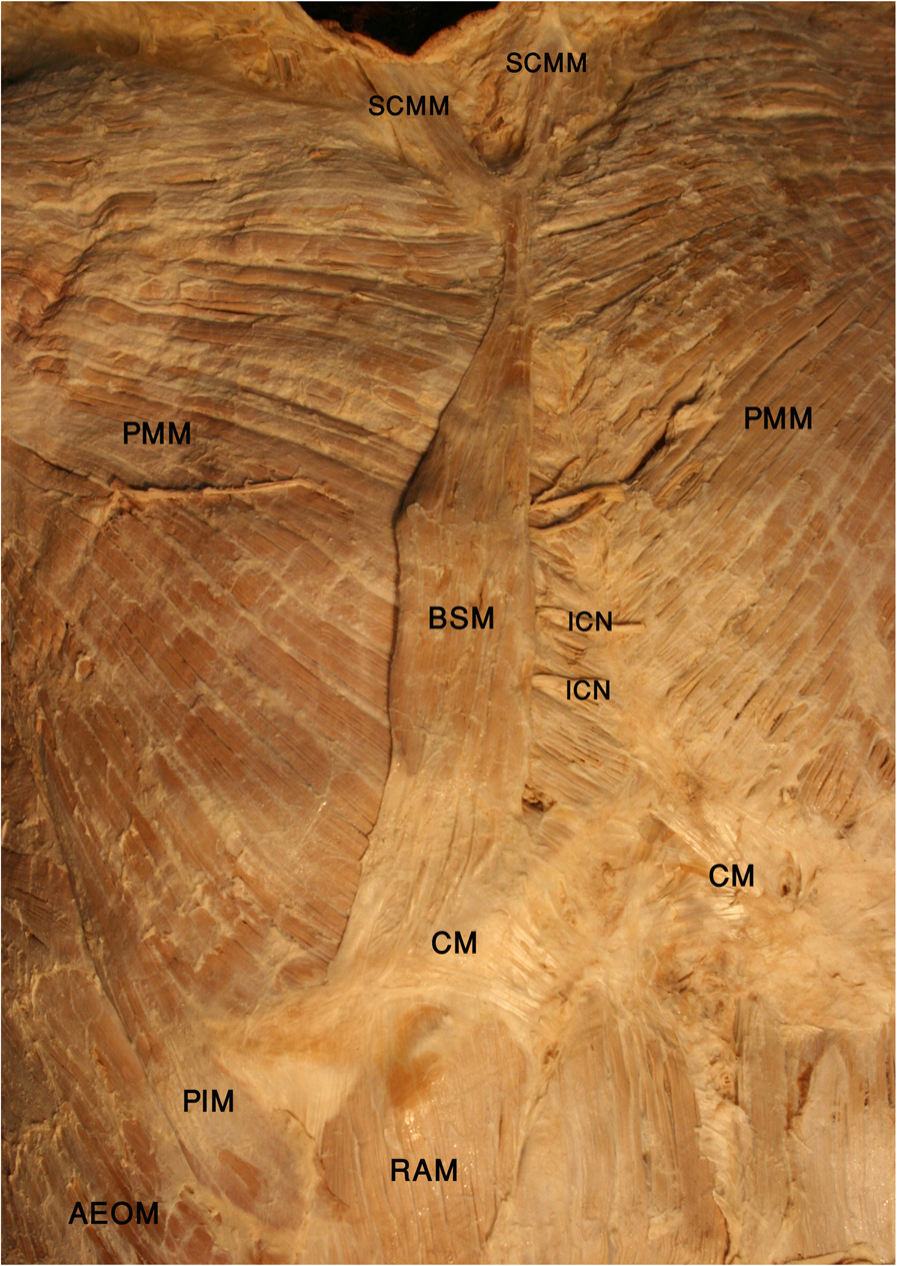
**The photograph shows the bicipital sternalis muscle (BSM) and the pectoralis inferior muscle (PIM) on the right side.** SCMM: sternocleidomastoid muscle, PMM: pectoralis major muscle, CM: costal margin, ICN: intercostal nerve, AEOM: abdominal external oblique muscle, RAM: rectus abdominis muscle.

**Figure 2:**
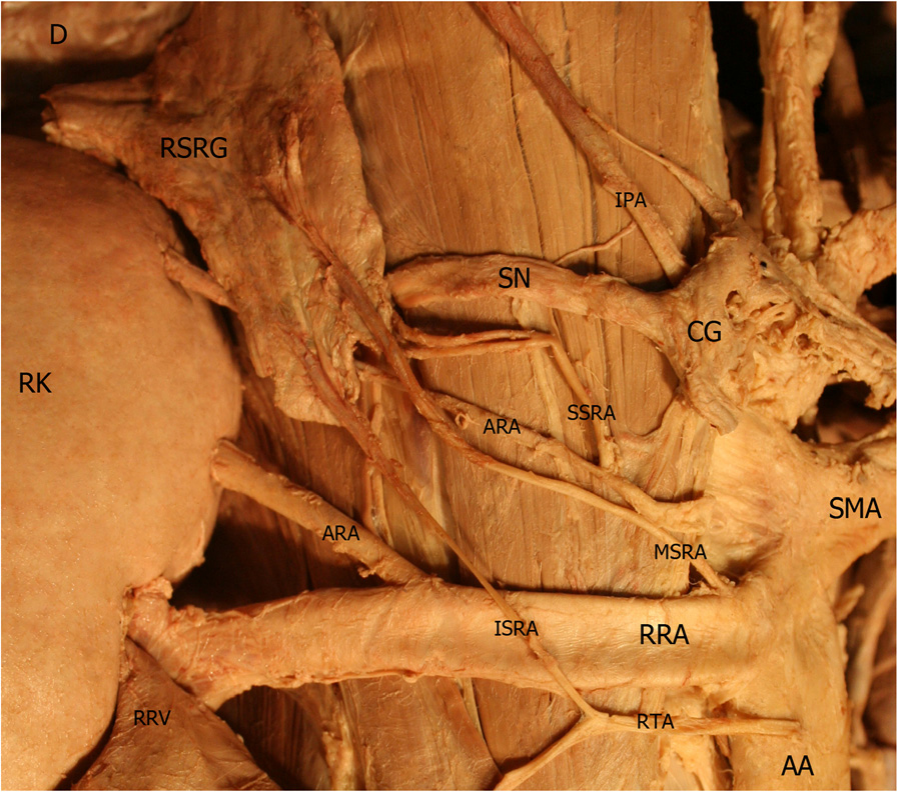
**Right retroperitoneal space dissection showing vascular variants of the right kidney, testis, and suprarenal gland.** RK: right kidney, RSRG: right suprarenal gland, AA: abdominal aorta, RRV: right renal vein, RRA: right renal artery, SMA: superior mesenteric artery, RTA: right testicular artery, IPA: inferior phrenic artery, SSRA: superior suprarenal artery, MSRA: middle suprarenal artery, ISRA: inferior suprarenal artery, D: diaphragm, CG: celiac ganglion, SN: splanchnic nerve.

During routine dissection of the chest wall in Iranian cadavers, an unusual case of an accessory thoracic muscle, the biceps sternalis, was encountered (Figure 1). The observed sternalis was a long, bicipital and strap-like muscle. It was localised anterior to the medial side of the right pectoralis muscle and its fascia. The upper third of the muscle was tendinous, the middle third was fleshy, and the lower third consisted of the aponeurosis. It presented with a slightly oblique orientation with respect to the sternal margin, which passed down toward the right parasternal half. This extra muscle was found between the anterior thoracic superficial fascia and the pectoral fascia. Its right and left borders were 15 and 14 cm, respectively. The maximum width of the muscle belly was 2.4 cm. It was observed that the sternalis lies in line with the left sternocleidomastoid muscle superiorly and the right rectus abdominis muscle inferiorly. The Y-shaped muscle tendon arose from the sternal head of the sternocleidomastoid muscles of both sides and connected to the muscle belly at the level of the sternal angle. Inferiorly, at the level of the xiphisternal joint, the muscle belly became a wide aponeurosis that attached to the right costal margin, rectus sheath and lower ribs. The muscle insertion was very close to the origin of the right rectus abdominis muscle, but there was no continuity between the muscle fibres of both. The anterior thoracic branches of the right intercostal nerves provided sternalis innervation. The remaining chest musculature exhibited normal morphology except for inferior fibres of the right pectoralis major or *pectoralis inferior*. These fibres crossed over the costal margin and inserted into the rectus sheath and linea semilunaris. Along with these muscular anomalies, as shown in Figure 2, vascular variants of the right kidney, testis, and suprarenal gland were also found [9]. Furthermore, multiple anomalies in the hepatobiliary system were also seen [10].

## Discussion

The sternalis muscle has been denoted variously [11,12]. A new nomenclature, “biceps sternalis”, can be used for this subtype, owing to its continuity with the sternocleidomastoid muscles of both sides and its longitudinal orientation in the anterior thoracic wall.

Origination from the sternum is one of the main criteria for a muscle to be categorised as sternalis [13]. The biceps sternalis in this case lacked any attachment to the sternum; however, its position in the subcutaneous plane, insertion and innervation support its categorisation as a sternalis muscle.

It seems that the sternalis muscle in this case has similarities to the cases presented in other studies, but to our knowledge, there is no identical case in the literature.

The unicep sternalis was first identified by Bradley et al., who described it in six women during the screening and diagnostic mammographic imaging of 32,000 women [14]. Arraez-Aybar et al. and Deepali et al., in cadaveric studies, observed a unilateral left-sided sternalis muscle [7,15]. This accessory muscle was also observed bilaterally [16,17].

The right-sided biceps sternalis, in this male case, had a regular flame shape along its longitudinal and parasternal course, which is similar to that of the muscle reported previously [14]. However, the contour of the unicep muscle in that report was shorter and irregular.

The pattern closest to this in the literature was mentioned in a report by Mehta et al. but with a different muscle origin, insertion and dimensions [18]. Another similar case was observed by Raikos et al., except in their case, they found that the muscle originated from the sternal head of the right sternocleidomastoid, crossed to the opposite side, and split into 2 parts that inserted into the left subcostal arch region [5].

Association of the sternalis muscle with several clinical conditions such as anencephaly and anomalies of the adrenal gland has been reported [19]. In this case, in addition to vascular anomalies of the adrenal gland, anomalies of the hepatobiliary system and anatomic variations of the renal and testicular vessels were also observed [9,10].

Two possible theories are suggested to explain the embryological origin of the sternalis muscle. The first is based on muscle innervations, and the second is based on muscle fibre arrangement. The innervation of the sternalis muscle depends on local signals [20]. Thus, varying innervations of the muscle are to be expected. These include the pectoral [19] or, less frequently, the intercostal nerves [16,17]. If the sternalis muscle is supplied by the pectoral nerves, it could originate from the pectoralis major. If the sternalis is supplied by the intercostal nerves, similar to this case, it could arise from the rectus abdominus muscle [13].

With regard to muscle fibre arrangement, the sternalis muscle may be an aberrant extension of the adjacent muscles or their blastemas. These muscles include the sternocleidomastoid muscle or the rectus abdominis muscle; however, the sternalis muscle is always superficial to the rectus abdominis and not continuous with it [11,21]. In addition, although the sternalis muscle is accompanied by a partial deficiency of the pectoralis major [22], the ipsilateral pectoralis major was observed to have additional muscle fibres (i.e., a pectoralis inferior) in this case. Furthermore, the sternalis was the downward continuation of the left sternocleidomastoid muscle. Hence, it is also possible that the present sternalis is a derivative of the sternocleidomastoid muscle.

According to these theoretical explanations, the sternalis muscle may have evolved from two different origins: superiorly from the sternocleidomastoid muscle and inferiorly from the rectus abdominis muscle. Finally, regarding muscle location and fibre direction, this biceps sternalis may assist in elevating the lower ribs.

## Conclusions

This case report illustrates two important concepts. First, an extra muscle may exhibit different morphologies, depending on gender and ethnic background. Second, a muscular anomaly may be associated with other vascular and visceral anomalies. An awareness of these types of anatomical variations is essential for cardiovascular, thoracic and general surgeons in order to avoid post-operative patient discomfort. Further research is needed to understand the association between muscular anomalies of the thoracic wall and congenital anomalies of the abdomen. Importantly, investigations into whether it is possible to anticipate associated anomalies based on the accidental observation of a particular anomalous structure would be informative for clinical practice.

## Consent

Written informed consent was obtained from the cadaver’s next of kin for publication of this Case report and any accompanying images. A copy of the written consent is available for review by the Editor-in-Chief of this journal.

## Abbreviations

CT: Computed tomography; MRI: Magnetic resonance imaging; BSM: Bicipital sternalis muscle; PIM: Pectoralis inferior muscle; SCMM: Sternocleidomastoid muscle; PMM: Pectoralis major muscle; CM: Costal margin; ICN: Intercostal nerve; AEOM: Abdominal external oblique muscle; RAM: Rectus abdominis muscle; RK: Right kidney; RSRG: Right suprarenal gland; AA: Abdominal aorta; RRV: Right renal vein; RRA: Right renal artery; SMA: Superior mesenteric artery; RTA: Right testicular artery; IPA: Inferior phrenic artery; SSRA: Superior suprarenal artery; MSRA: Middle suprarenal artery; ISRA: Inferior suprarenal artery; D: Diaphragm; CG: Celiac ganglion; SN: Splanchnic nerve.

## Competing interests

The author declares that he has no competing interests.

## Authors’ contributions

SHA obtained written informed consent from the cadaver’s next of kin, performed the cadaver dissection, photographed the anomalous structures, reviewed the literature, and acquired data. He also drafted the manuscript and read and approved the final manuscript for publication.
